# A rare tumour of the vulva: a case report of a vulva angioneurofibroma hamartoma in a Cameroonian woman

**DOI:** 10.11604/pamj.2013.15.115.2597

**Published:** 2013-07-29

**Authors:** Florent Fouelifack Ymele, Efuetnkeng Bechem, Philip Nana Njotang, Charlette Nangue, Jeanne Hortence Fouedjio, Sadjoli Damtheou, Robinson Mbu Enoh

**Affiliations:** 1 Obstetrics and Gynecology Unit of Yaounde Central Hospital, Cameroon; 2 Research, Education and Health Development Associates Group (REHDAG) Dschang, Cameroon; 3 Department of Obstetrics and Gynaecology, Faculty of Medicine and Biomedical Sciences University of Yaoundé 1, Cameroon; 4 Pathology unit of Yaounde Central Hospital, Cameroon

**Keywords:** Tumour, vulva, ngioneurofibroma hamartoma

## Abstract

We present the case of a rare vulva tumour, in a 33 years Cameroonian old woman and managed in Obstetrics and Gynecology Unit of Yaoundé Central Hospital in Cameroon. It was a painless pedunculated vulva tumour which developed over a period of six months. This gigantic rapidly growing tumour, was treated with simple surgical resection. After surgical resection, histology confirmed an angioneurofibroma hamartoma. There has been no recurrence and presently the patient is symptom-free.

## Introduction

A hamartoma is a benign lesion composed of various native tissues, but growing in a disorganized manner. It is not considered a malignant growth, as it grows at the same rate as surrounding cells [1]. It may occur in different parts of the body. The vulva is an organ composed of squamous and glandular epithelium, and therefore presents several architectural benign lesions [2]. We present the case of a rare vulva hamartoma in a young Cameroonian woman. This is a rare affection of a giant tumour affecting the social well being of the patient and treated by a simple surgical excision.

## Patient and observation

A 33 year old, G3P2012 (last child 2 years of age), consulted in the outpatient department of Obstetrics and Gynecology Unit of Yaounde Central Hospital in Cameroon two years ago, for a growth in her vulva. This started about 6 months before, and was painless and rapidly growing. She did not present any other symptom. She had consulted several tradi-practitioners but there was no regression.

**Figure 1:**
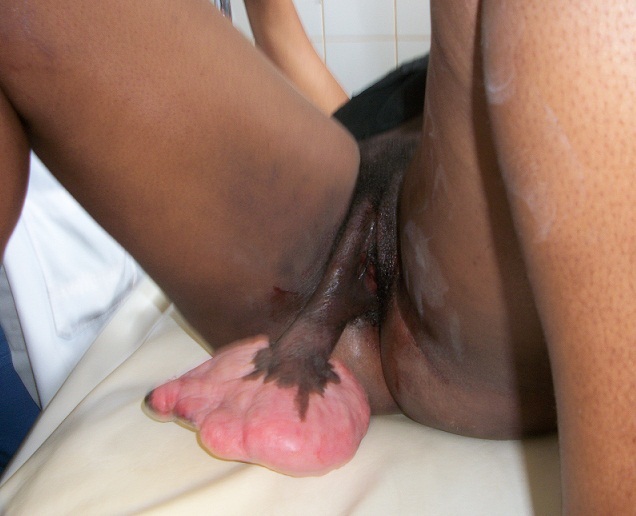
Macroscopic aspect of the tumor before surgery showing the pedonculated tumor fixed on the right labia majora of the vulvae

On physical examination, her general condition was satisfactory, and she presented a pedunculated tumour in her vulva. The tumour was implanted in her right labia majora, a pedicle measuring 17cm length and 5cm diameter, and a base of 25cm diameter (Figure 1). The rest of the physical examination was not contributive.

We thought it was a vulva tumour probably of embryologic origin and to rule out an acrochordon (fibroepithelial polyp of the vulva), a lymphadenoma and fibromyoma of the vulva. She was screened HIV negative and other biological tests were normal.

**Figure 2:**
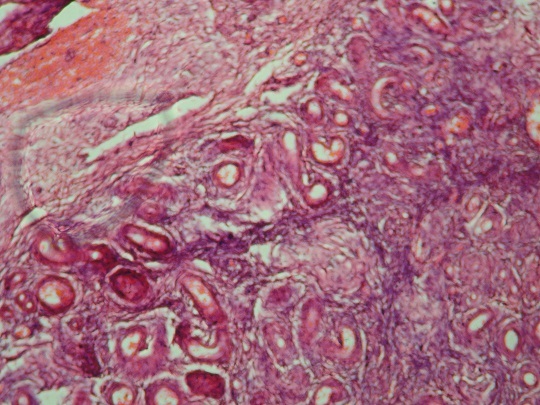
Histology of the tumor showing vessels, nervus tissue and connective tissue

The tumour was excised under general anaesthesia, and the patient discharged the following day on sits baths. On gross examination of the tumour, it was a tumour of 25cm diameter, with a depigmented surface and a mucosa. The pedicle was retracted and 10 cm long with a pigmented surface. There were blood vessels in the pedicle. Histology of the tumour reported a vulva hamartoma of the angioneurofibroma type (Figure 2). Two years after the surgery she is alive and free of recurrence.

## Discussion

A hamartoma is a benign, focal malformation that resembles a neoplasm in the tissue of its origin. This is not a malignant tumor, and it grows at the same rate as the surrounding tissues. It is composed of tissue elements normally found at that site, but which are growing in a disorganized manner [3]. Albrecht in 1904 was the first to use the term hamartoma. He described these tumours as developmental tumour-like malformations, in which the normal elements of the organ are abnormally represented, in quality, arrangement, or degree of differentiation or all of the three [4].

A hamartoma may consist of a single type of cell, a particular type of tissue or a mixture of tissues. They occur in many different parts of the body and are most often asymptomatic. These benign tumor-like tissues over growths may be congenital or develop later in life. Two types of hamartomas are distinguished: infantile and adult types. The infantile type is a true hamartoma, but that the adult type is referred to as a mixed tumor [5].

Hamartomas of the vulvae are rare benign lesions [6]. Very few cases have been reported in literature. They are named following the tissue identified.

Our patient being a 33 year old presented an adult mixed type. The tumour showed an area of well-demarcated interwoven collagen bundles arranged around blood vessels and nerve fibres (Figure 2), and therefore the name angioneurofibroma.

Differential diagnosis of such a tumor can be made with an acrochordon which is often multiple and appearing as soft, pedunculated, brown, tan, or skin-colored lesions (0.2-1.5 cm in diameter), and can particularly be found in the inguinal folds of obese and/or diabetic patients, or with a lymphangioma which is usually detected early in infancy on the labia minora or majora as an asymptomatic, raised, compressible, doughy mass, sometimes showing multiple clustered, superficial, thin-walled, translucent, and persistent pseudovesicles filled with clear fluid that may progressively grow over time.

Treatment of hamartomas consists of surgical removal of the tumor. The patient should be closely followed for recurrences, which may occur if only partial resection of tumor was done [7]. Our patient benefited from a surgical excision with no recurrence.

Complications of the tumor includes: pain, infection, social misfits, and a less than 1% risk of cancerisation in the long term [2]. Some complications following treatment are: recurrence, bleeding, and poor scarring rendering coitus difficult.

## Conclusion

The interest of this case is to recall the existence of vulva hamartomas. These tumors can be giant but with simple surgical excision as treatment.
